# Differentially expressed genes related to major depressive disorder and antidepressant response: genome-wide gene expression analysis

**DOI:** 10.1038/s12276-018-0123-0

**Published:** 2018-08-03

**Authors:** Hye In Woo, Shinn-Won Lim, Woojae Myung, Doh Kwan Kim, Soo-Youn Lee

**Affiliations:** 1 Department of Laboratory Medicine, Samsung Changwon Hospital, Sungkyunkwan University School of Medicine, Changwon, Korea; 2 SAIHST, Sungkyunkwan University School of Medicine, Samsung Medical Center, Seoul, Korea; 3 Department of Neuropsychiatry, Seoul National University Bundang Hospital, Seongnam, Korea; 4 Department of Psychiatry, Samsung Medical Center, Sungkyunkwan University School of Medicine, Seoul, Korea; 5 Department of Clinical Pharmacology & Therapeutics, Samsung Medical Center, Seoul, Korea; 6 Department of Laboratory Medicine & Genetics, Samsung Medical Center, Sungkyunkwan University School of Medicine, Seoul, Korea

## Abstract

Treatment response to antidepressants is limited and varies among patients with major depressive disorder (MDD). To discover genes and mechanisms related to the pathophysiology of MDD and antidepressant treatment response, we performed gene expression analyses using peripheral blood specimens from 38 MDD patients and 14 healthy individuals at baseline and at 6 weeks after the initiation of either selective serotonin reuptake inhibitor (SSRI) or mirtazapine treatment. The results were compared with results from public microarray data. Seven differentially expressed genes (DEGs) between MDD patients and controls were identified in our study and in the public microarray data: *CD58*, *CXCL8*, *EGF*, *TARP*, *TNFSF4*, *ZNF583*, and *ZNF587*. *CXCL8* was among the top 10 downregulated genes in both studies. Eight genes related to SSRI responsiveness, including *BTNL8*, showed alterations in gene expression in MDD. The expression of the *FCRL6* gene differed between SSRI responders and nonresponders and changed after SSRI treatment compared to baseline. In evaluating the response to mirtazapine, 21 DEGs were identified when comparing MDD patients and controls and responders and nonresponders. These findings suggest that the pathophysiology of MDD and treatment response to antidepressants are associated with a number of processes, including DNA damage and apoptosis, that can be induced by immune activation and inflammation.

## Depression: genes involved in illness and drug response

Differences in the expression of several genes before and after different antidepressant treatments were found in patients with major depressive disorder (MDD), and may help identify patients most likely to benefit from specific drugs. Researchers in South Korea led by Doh Kwan Kim and Soo-Youn Lee at Samsung Medical Center, Seoul, examined gene expression across the 28,869 genes in 38 patients with MDD and 14 healthy individuals. They also validated their findings using existing databases of gene expression in patients with MDD and healthy controls. The research suggests that genes involved in the immune response and inflammation are significantly alternated in MDD and are predictable in which patients respond well to antidepressants. These findings may help develop new approaches to antidepressant therapies, and assist tailoring of treatment to the specific needs of different patients.

## Introduction

Major depressive disorder (MDD) is a major burden in healthcare; worldwide, 12% of individuals suffer from MDD^1,2^. MDD is considered to develop as a consequence of environmental influences on genetic predispositions, but a definite pathogenesis of MDD remains obscure^3,4^. There have been several hypotheses for the pathogenesis of MDD, including alterations in neurotrophins, the neuroendocrine and neuroimmune systems, and molecules involved in brain neurotransmission, including monoamines and glutamate, as well as epigenetic mechanisms^4–9^. Other hypotheses include alterations in immune and inflammatory responses, oxidative stress, mitochondrial dysfunction, and disruption of DNA damage responses, and biomarkers related to these mechanisms have been proposed^10–17^. Although there are environmental factors known to be related to the development of MDD, such as stressful events in childhood, there is still no reliable biomarker that can explain the development of MDD or differences between MDD patients and healthy individuals.

For pharmacotherapy of MDD, many second-generation antidepressants are used, such as selective serotonin reuptake inhibitors (SSRIs), serotonin and norepinephrine reuptake inhibitors (SNRIs), atypical antidepressants, and serotonin modulators. SSRIs potentiate serotonin (5-HT) by inhibiting its neuronal uptake pump. Some SSRIs also have minor noradrenaline and dopamine reuptake inhibitory properties^18^. Mirtazapine has a dual mode of action, antagonizing the adrenergic α_2_-autoreceptors and α_2_-heteroreceptors as well as by blocking 5-HT_2_ and 5-HT_3_ receptors^19^. At least one-third of patients treated with second-generation antidepressants do not achieve response^20,21^. Although there is no evident difference in overall efficacy among second-generation antidepressants, individuals vary widely in their response to specific antidepressant treatments^20–22^. To predict responses to antidepressants and to choose the appropriate treatment for each individual, the discovery of biomarkers related to therapeutic efficacy is urgently required. Thus far, there have been few studies to identify biomarkers that could predict therapeutic response in MDD, and there is no absolute predictor to help guide the selection of antidepressants^23–31^.

RNA is the immediate expression product of genes and better reflects the current functional status of the biologic system than does DNA. Recently, a number of gene expression profiling studies aiming to discover genetic markers related to the pathogenesis of MDD and antidepressant treatment response using RNA from postmortem brain tissues and peripheral blood have been published^32–41^. Most of these studies focused on alterations in gene expression in MDD patients compared with healthy controls, while a small number of studies focused on gene expression differences and changes according to antidepressant treatment and responsiveness^27–31^. Transcriptomic studies on antidepressant treatment are insufficient considering the variety of antidepressants in use, and previous studies on treatment response have been limited to patients treated with citalopram or cognitive behavioral therapy or did not consider the type of antidepressant used^27–31^.

In this study, we performed gene expression profiling of peripheral blood samples to assess differences between MDD patients and healthy individuals. We also evaluated differences in gene expression according to treatment response and changes after antidepressant treatment. Our findings contribute to the understanding of the pathophysiologic derangement in MDD patients and the mechanisms and genes involved in antidepressant treatment response at the RNA level.

## Materials and Methods

### Patients

A total of 38 Korean patients with MDD who fulfilled the Diagnostic and Statistical Manual of Mental Disorders, Fourth Edition (DSM-IV) criteria for MDD without psychotic features, as well as 14 healthy individuals, were included in this study. Diagnoses were confirmed by a board-certified psychiatrist based on an initial clinical interview, followed by a structured research assessment with the Samsung Psychiatric Evaluation Schedule^42^, which includes the Structured Clinical Interview for DSM-IV^42,43^. A minimum baseline 17-item Hamilton Depression Rating Scale (HAM-D) score of 15 was required^44^. Study participants were excluded in cases of pregnancy, significant medical conditions, unstable psychiatric features (e.g., suicide attempt in the current episode), history of alcohol or drug dependence, seizures, head trauma with loss of consciousness, neurological illness, or a concomitant Axis I psychiatric disorder. No patient had received psychotropic medication for the current. Patients were treated with 10–30 mg/day escitalopram, 20 mg/day paroxetine, 100 mg/day for sertraline, or 15–45 mg/day mirtazapine. To monitor compliance, we routinely checked the plasma concentrations of antidepressants and performed pill counts at every clinic visit. Therapeutic response was defined as a 50% or greater decrease in HAM-D score by 6 weeks after the initiation of antidepressant treatment. This study was approved by the Samsung Medical Center Institutional Review Board. Written informed consent was obtained by all participants.

### Gene expression profiling

Peripheral blood samples were collected from patients at baseline and 6 weeks after the initiation of antidepressant therapy and from healthy individuals at baseline only. Blood was drawn between 0800 and 1000 h. Total RNA was extracted from peripheral blood specimens using TRIzol^®^ Reagent (Life Technologies, Carlsbad, CA, USA) according to the manufacturer’s instructions. Isolated RNA was stored at −70 °C. For gene expression profiling, we used GeneChip^®^ Human Gene 1.0 ST Arrays (Affymetrix, Santa Clara, CA, USA), which offer whole-transcript coverage of 28,869 genes. The arrays were scanned with a GeneChip^®^ Scanner 3000 7G (Affymetrix), and probe cell intensity data were generated with GeneChip^®^ Command Console^®^ software (Affymetrix).

### Data analyses

The statistical analysis software package R 3.1.2 was used for data analyses^45^. We performed background adjustment and quantile normalization using the robust multiarray average (RMA) algorithm in the affy R package^46^. After normalization, five samples with poor normalized unscaled standard error (NUSE) and spatial defects were excluded, including one 6-week sample from a patient who responded to escitalopram, one baseline sample from a patient who did not respond to mirtazapine, and three samples from healthy individuals. We classified patients by antidepressant use into mirtazapine and SSRIs (escitalopram, paroxetine, or sertraline) groups and performed further statistical analysis according to this classification. We performed hierarchical cluster analysis using Ward’s hierarchical agglomerative clustering method for two groups^47^. To identify differentially expressed genes (DEGs) between MDD patients and healthy individuals and between responders and nonresponders to antidepressants, we performed the moderated *t*-test with the limma R package^48^. Pairwise, within-subject comparisons of gene expression profiles at baseline and at 6 weeks after antidepressant treatment were performed with paired *t*-tests. Genes with both *p*-values <0.05 and an absolute FC > 1.2 in each specific comparison analysis were considered statistically significant. Corrected *p*-values were obtained using the Benjamini–Hochberg false discovery rate (FDR) approach. Gene ontology (GO) and pathway enrichment analyses for genes adhering to these criteria were performed using DAVID tools^49,50^. We also performed a network analysis according to the biological interactions of DEGs in each comparison. We used a public database, Reactome^51,52^, and reformatted it using Pathway Commons^53^. Cytoscape was used as a visualization tool to generate the network^54^. To validate our study results, we used an RNA microarray data set from a public database, the Gene Expression Omnibus (GEO). We investigated replicates among comparisons that were performed in our study. The public microarray data were obtained from leukocytes from eight MDD patients and eight controls (http://www.ncbi.nlm.nih.gov/geo/query/acc.cgi?acc = GSE32280). Using these data sets, we extracted DEGs and GO terms in the same manner as was used for our own data and correlated them with the results from our study. As another method to validate the results, we compared our findings with the results from our recent study that addressed the evaluation of cytokines between healthy individuals and MDD patients^55^.

## Results

### Patient characteristics

Table 1 summarizes the baseline clinical characteristics of MDD patients and healthy individuals. Overall, SSRI- and mirtazapine-treated patients and healthy individuals had no statistically significant differences in baseline clinical characteristics. Responders and nonresponders also did not differ at baseline in any of the measured variables.

**Table 1:** Characteristics of healthy controls and major depressive disorder patients at baseline

Characteristics	SSRIs		Mirtazapine		Control	*P*
	*R*	*NR*	R	NR		
*N*	14	6	8	10	11	
Age, median (Q1-Q3), y	70.5 (61.3–73.0)	63.0 (58.0–71.8)	70.0 (63.5–74.3)	65.5 (56.8–69.5)	69.0 (66.0–75.5)	0.385^a^
Sex, M:F	2:12	1:5	1:7	3:7	2:9	0.797^b^
HAM-D score, median (Q1-Q3)	17.5 (16.3–19.0)	20(17.8–20.8)	20 (17.5–21.3)	22.5 (19.0–23.8)	–	0.262^a^
Duration of current episode, median (Q1-Q3), wks	2.0 (1.3–8.8)	3.0 (2.3–9.8)	7.0 (2.8–13.3)	3.5 (2.0–5.0)	–	0.312^a^
Antidepressant used, n (range of dose, mg/kg)						0.521^c^
Escitalopram	11 (0.2–0.4)	6 (0.2–0.4)	–	–	–	0.794^d^
Paroxetine	2 (0.4)	0	–	–	–	
Sertraline	1 (1.5)	0	–	–	–	
Mirtazapine	–	–	8 (0.4–0.8)	10 (0.3–1.0)	–	0.253^d^

*NR* nonresponders, *Q*1 lower quartile, *Q*3 upper quartile, *R* responders, *SSRIs* selective serotonin reuptake inhibitors

^a^*P*-value from Kruskal–Wallis test

^b^*P*-value from Fisher’s exact test

^c^*P*-value from Fisher’s exact test to evaluate the difference in SSRIs used between responders and nonresponders

^d^*P*-value from Mann–Whitney U test

### DEGs in MDD

**Fig. 1:**
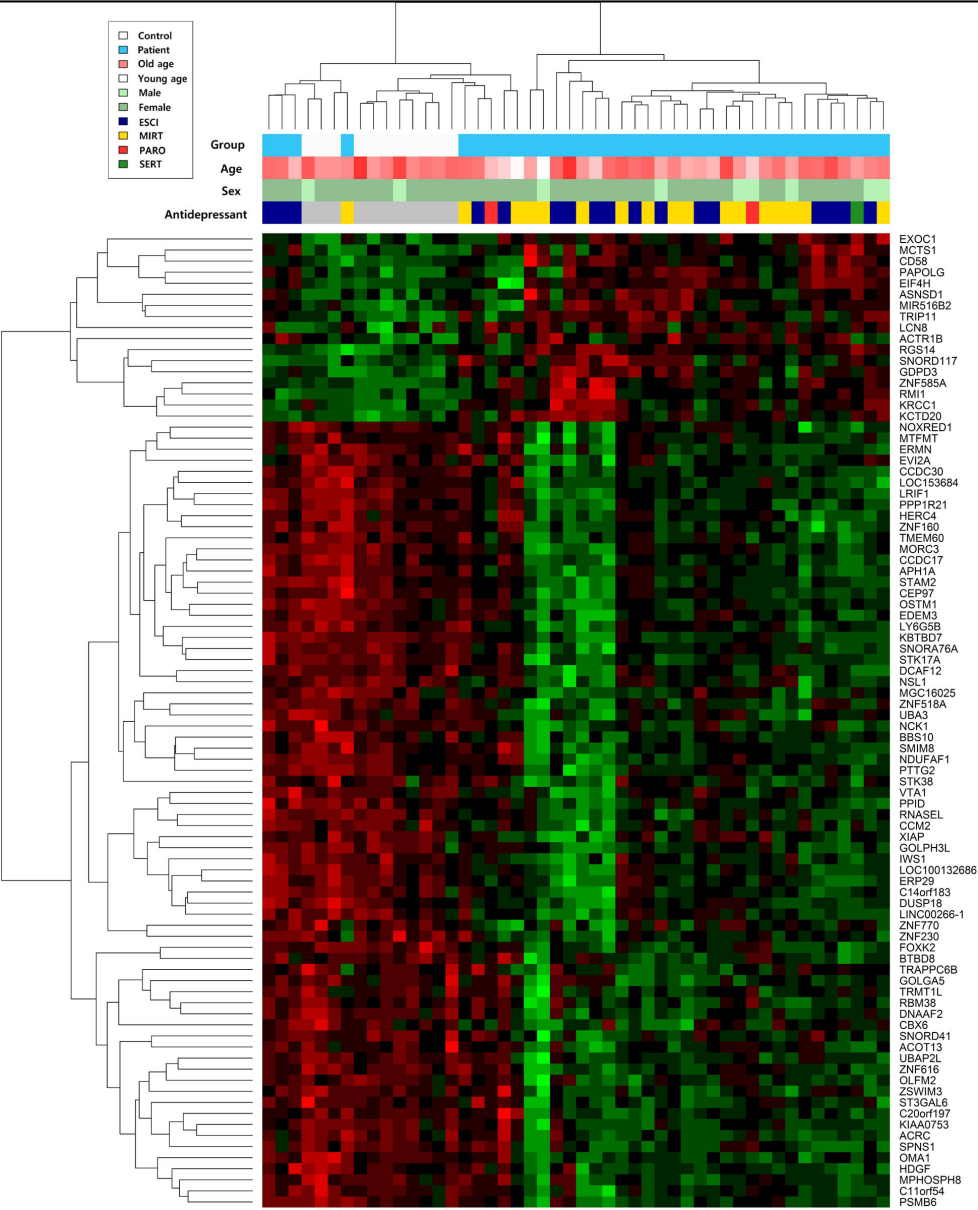
Hierarchical clustering of 88 genes that were differentially expressed in major depressive disorder patients at baseline and controls; the criteria were both false discovery rate (FDR)-corrected *p*-value < 0.1 and absolute fold change (FC) > 1.2. Red indicates upregulated genes, green indicates downregulated genes, and black indicates genes with similar expression levels. *Esci* escitalopram, *Mirt* mirtazapine, *Paro* paroxetine, *Sert* sertraline

When comparing patients at baseline and controls, the expression levels of 476 genes differed based on the criteria of uncorrected *p*-value < 0.05 and fold change (FC) > 1.2. Functional annotation of these genes using GO and pathway enrichment analyses indicated that 32 GO biological process terms and four pathways were overrepresented, with the criteria of an uncorrected *p*-value < 0.05 (Supplementary Table 1). Many of the enriched GO terms and pathways are known to be involved in immune and inflammatory responses and apoptosis. Hierarchical clustering demonstrated that MDD patients at baseline were distinct from healthy controls, and this separation was not dependent on age, sex, or antidepressant use (Fig. 1). The top 10 downregulated and upregulated genes according to FC value were identified and are listed in Supplementary Table 2.

**Fig. 2:**
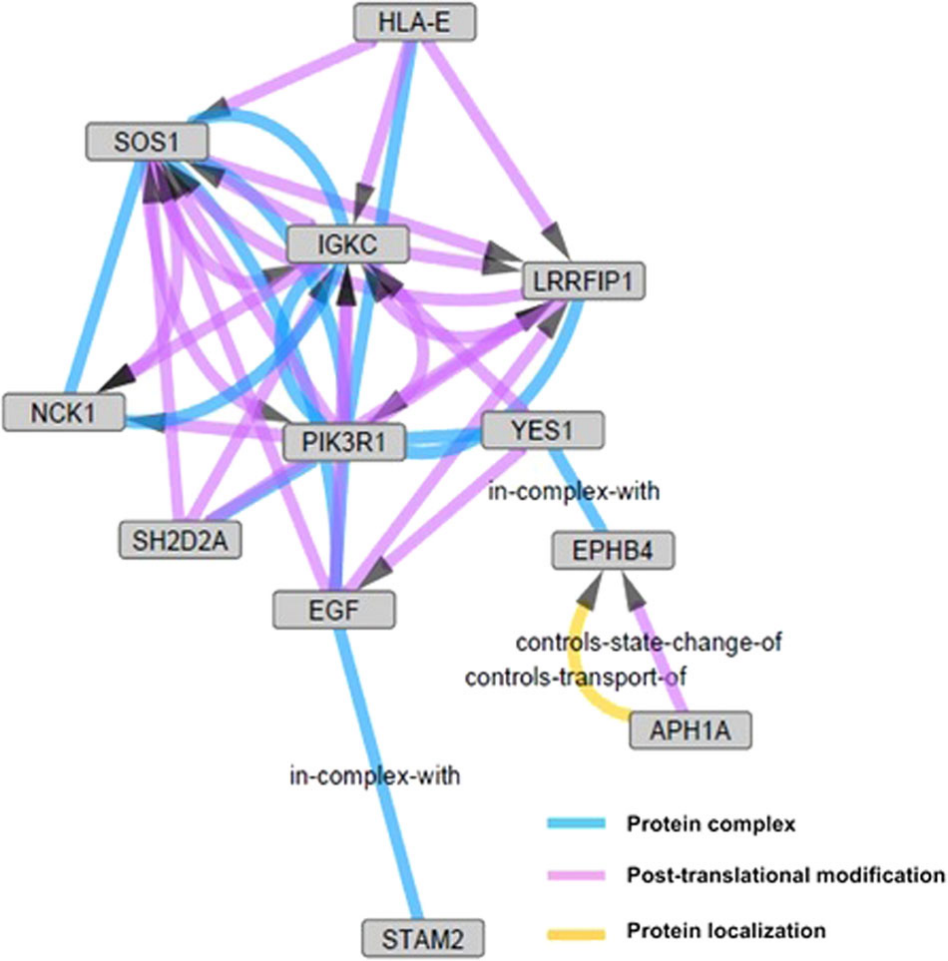
Gene network of DEGs between major depressive disorder patients and healthy controls. Twelve genes related to signal transduction and the immune system are involved in this network with an uncorrected *p*-value < 0.05. *APH1A*, aph-1 homolog A, gamma secretase subunit; *EGF*, epidermal growth factor; *EPHB4*, EPH receptor B4; *HLA-E*, major histocompatibility complex class I E; *IGKC*, immunoglobulin kappa constant; *LRRFIP1*, leucine-rich repeat (in FLII) interacting protein 1; *NCK1*, NCK adaptor protein 1; *PIK3R1*, phosphoinositide-3-kinase regulatory subunit 1; *SH2D2A*, SH2 domain containing 2A; *SOS1*, SOS Ras/Rac guanine nucleotide exchange factor 1; *STAM2*, signal transducing adaptor molecule 2; *YES1*, YES proto-oncogene 1, Src family tyrosine kinase

In the analysis of public microarray data from eight MDD patients and eight controls, 375 DEGs were identified. Following a comparison with our study results, seven DEGs between MDD patients and controls were identified in both analyses: *CD58*, *CXCL8*, *EGF*, *TARP*, *TNFSF4*, *ZNF583*, and *ZNF587* (Table 2). In both studies, the *CXCL8*, *EGF*, and *TNFSF4* genes were downregulated in MDD patients, and the other genes were upregulated in MDD patients. Among these genes, *CXCL8* was among the top 10 downregulated genes in both studies, and the level of IL-8, which is encoded by the *CXCL8* gene, was decreased in MDD patients compared with healthy individuals in our previous cytokine study^55^. In GO enrichment and pathway analyses, seven GO biological process terms were identified as common to both studies: cell activation (GO:0001775), immune response (GO:0006955), cell cycle (GO:0007049), enzyme-linked receptor protein signaling pathway (GO:0007167), protein kinase cascade (GO:0007243), phosphorylation (GO:0016310), and lymphocyte activation (GO:0046649). Network analysis using 476 DEGs generated 11 networks. Most of the networks involved a small number of genes, i.e., five or fewer. Only one network, related to signal transduction and the immune system, involved more than five genes (Fig. 2).

**Table 2:** Differentially expressed genes in major depressive disorder patients compared to healthy controls in our data and public microarray data

Gene symbol	Full gene name	Our study			Public microarray data		
		FC^a^	*P*	Corrected *P*	FC^a^	*P*	Corrected *P*
*CD58*	CD58 molecule	1.24	6.09 × 10^−4^	9.29 × 10^−2^	1.25	1.55 × 10^−2^	1.00 × 10^0^
*CXCL8*	Chemokine (C-X-C motif) ligand 8	0.71	3.45 × 10^−2^	3.93 × 10^−1^	0.33	3.70 × 10^−2^	1.00 × 10^0^
*EGF*	Epidermal growth factor	0.79	4.98 × 10^−2^	4.50 × 10^−1^	0.71	9.82 × 10^−3^	1.00 × 10^0^
*TARP*	TCR gamma alternate reading frame protein	1.33	4.06 × 10^−2^	4.18 × 10^−1^	1.44	8.17 × 10^−3^	1.00 × 10^0^
*TNFSF4*	Tumor necrosis factor superfamily member 4	0.74	1.82 × 10^−2^	3.18 × 10^−1^	0.66	3.63 × 10^−2^	1.00 × 10^0^
*ZNF583*	Zinc finger protein 583	1.24	1.76 × 10^−3^	1.38 × 10^−1^	1.23	3.85 × 10^−2^	1.00 × 10^0^
*ZNF587*	Zinc finger protein 587	1.22	1.58 × 10^−2^	3.05 × 10^−1^	1.35	5.82 × 10^−3^	1.00 × 10^0^

*FC* fold change

^a^Fold changes in patients compared to controls

### Genes related to treatment response to SSRIs

When comparing gene expression at baseline between SSRI responders and nonresponders, 222 DEGs were identified. GO and pathway enrichment analyses of these genes identified 33 GO terms and three pathways (Supplementary Table 1). Similar to the analysis between MDD patients and controls, GO terms involved in immune response and apoptosis were the most enriched. To identify changes in gene expression related to SSRI treatment responsiveness, we compared gene expression at 6 weeks after treatment to that at baseline. Twenty-eight DEGs were identified only in responders. According to their FC values, the top 10 downregulated and upregulated genes were identified and are listed in Supplementary Table 3.

Among the identified DEGs, nine genes were repeatedly identified, i.e., identified in two comparisons: *HSPH1* and *IGKC* in comparisons between MDD patients and controls and between responders and nonresponders to SSRIs; *BTNL8*, *KATNBL1*, *LYVE1*, *MIR15A*, *PTCH2*, and *SCARNA17* in comparisons between MDD patients and controls and between baseline and 6 weeks after SSRI treatment; and *FCRL6* in comparisons between responders and nonresponders to SSRIs and between baseline and 6 weeks after SSRI treatment (Table 3). Of these genes, *BTNL8* and *FCRL6* were among the top 10 genes in both comparisons.

**Table 3:** Differentially expressed genes repeatedly (≥2) identified in the three comparisons (C vs. P, R vs. NR, and Pre vs. Post)

Gene symbol	Full gene name	Comparison	FC^a^	*P*	Corrected *P*	Comparison	FC^a^	*P*	Corrected *P*
Responsiveness to SSRIs									
*FCRL6*	Fc receptor-like 6	R vs. NR	1.48	3.54 × 10^−2^	4.78 × 10^−1^	Pre vs. Post	1.21	1.72 × 10^−3^	6.94 × 10^−2^
*HSPH1*	Heat shock 105 kDa/110 kDa protein 1	R vs. NR	1.41	2.36 × 10^−2^	4.45 × 10^−1^	C vs. P	1.32	1.24 × 10^−2^	2.82 × 10^−1^
*IGKC*	Immunoglobulin kappa constant	R vs. NR	1.64	6.30 × 10^−3^	3.95 × 10^−1^	C vs. P	0.76	4.29 × 10^−2^	4.25 × 10^−1^
*BTNL8*	Butyrophilin-like 8	Pre vs. Post	0.81	5.54 × 10^−3^	1.06 × 10^−1^	C vs. P	1.59	1.63 × 10^−3^	1.33 × 10^−1^
*KATNBL1*	Katanin p80 subunit B-like 1	Pre vs. Post	0.83	1.02 × 10^−3^	6.12 × 10^−2^	C vs. P	1.38	2.02 × 10^−3^	1.41 × 10^−1^
*LYVE1*	Lymphatic vessel endothelial hyaluronan receptor 1	Pre vs. Post	0.83	9.09 × 10^−4^	5.95 × 10^−2^	C vs. P	1.28	3.56 × 10^−2^	3.97 × 10^−1^
*MIR15A*	MicroRNA 15a	Pre vs. Post	0.81	2.82 × 10^−2^	2.05 × 10^−1^	C vs. P	1.50	1.36 × 10^−2^	2.91 × 10^−1^
*PTCH2*	Patched 2	Pre vs. Post	1.21	4.57 × 10^−3^	9.84 × 10^−2^	C vs. P	0.60	7.97 × 10^−4^	1.04 × 10^−1^
*SCARNA17*	Small Cajal body-specific RNA 17	Pre vs. Post	1.21	1.08 × 10^−3^	6.15 × 10^−2^	C vs. P	0.78	1.89 × 10^−2^	3.23 × 10^−1^
Responsiveness to mirtazapine									
*GPAT3*	Glycerol-3-phosphate acyltransferase 3	R vs. NR	1.39	4.55 × 10^−2^	5.44 × 10^−1^	C vs. P	1.32	1.86 × 10^−2^	3.20 × 10^−1^
*ANKRD55*	Ankyrin repeat domain 55	R vs. NR	1.33	2.08 × 10^−2^	5.23 × 10^−1^	C vs. P	1.20	4.12 × 10^−2^	4.19 × 10^−1^
*BRCA1*	Breast cancer 1	R vs. NR	1.26	3.36 × 10^−2^	5.33 × 10^−1^	C vs. P	1.21	1.06 × 10^−2^	2.63 × 10^−1^
*C11orf54*	Chromosome 11 open reading frame 54	R vs. NR	1.22	4.65 × 10^−2^	5.48 × 10^−1^	C vs. P	1.30	2.98 × 10^−4^	7.51 × 10^−2^
*C20orf197*	Chromosome 20 open reading frame 197	R vs. NR	1.22	2.16 × 10^−2^	5.24 × 10^−1^	C vs. P	1.24	5.21 × 10^−4^	8.60 × 10^−2^
*CC2D2B*	Coiled-coil and C2 domain containing 2B	R vs. NR	1.27	4.68 × 10^−2^	5.48 × 10^−1^	C vs. P	1.26	3.14 × 10^−2^	3.80 × 10^−1^
*CEP63*	Centrosomal protein 63 kDa	R vs. NR	1.37	4.36 × 10^−2^	5.43 × 10^−1^	C vs. P	1.27	2.93 × 10^−2^	3.72 × 10^−1^
*DUSP18*	Dual specificity phosphatase 18	R vs. NR	1.22	3.46 × 10^−2^	5.34 × 10^−1^	C vs. P	1.28	3.78 × 10^−4^	8.15 × 10^−2^
*FRMD4B*	FERM domain containing 4B	R vs. NR	0.76	2.98 × 10^−2^	5.26 × 10^−1^	C vs. P	0.83	3.58 × 10^−2^	3.98 × 10^−1^
*GPSM2*	G-protein signaling modulator 2	R vs. NR	1.34	2.58 × 10^−2^	5.26 × 10^−1^	C vs. P	1.21	4.06 × 10^−2^	4.18 × 10^−1^
*IGF2BP2*	Insulin-like growth factor 2 mRNA binding protein 2	R vs. NR	0.79	2.77 × 10^−2^	5.26 × 10^−1^	C vs. P	0.82	7.66 × 10^−3^	2.30 × 10^−1^
*KIAA0753*	KIAA0753	R vs. NR	1.20	4.46 × 10^−2^	5.43 × 10^−1^	C vs. P	1.30	9.02 × 10^−5^	5.20 × 10^−2^
*LIMS1*	LIM and senescent cell antigen-like domains 1	R vs. NR	0.73	2.18 × 10^−2^	5.24 × 10^−1^	C vs. P	0.82	4.14 × 10^−2^	4.21 × 10^−1^
*LOC100132686*	Uncharacterized LOC100132686	R vs. NR	1.22	7.49 × 10^−3^	5.05 × 10^−1^	C vs. P	1.26	5.83 × 10^−5^	4.84 × 10^−2^
*MPHOSPH8*	M-phase phosphoprotein 8	R vs. NR	1.22	1.37 × 10^−2^	5.07 × 10^−1^	C vs. P	1.26	1.78 × 10^−4^	5.99 × 10^−2^
*PLXND1*	Plexin D1	R vs. NR	0.78	2.30 × 10^−2^	5.26 × 10^−1^	C vs. P	0.83	1.21 × 10^−2^	2.81 × 10^−1^
*PPP1R21*	Protein phosphatase 1 regulatory subunit 21	R vs. NR	1.20	3.36 × 10^−2^	5.33 × 10^−1^	C vs. P	1.28	9.75 × 10^−5^	5.20 × 10^−2^
*SLA2*	Src-like-adaptor 2	R vs. NR	0.74	3.70 × 10^−2^	5.37 × 10^−1^	C vs. P	0.80	2.40 × 10^−2^	3.48 × 10^−1^
*TOPORS*	Topoisomerase I binding, arginine/serine-rich, E3 ubiquitin protein ligase	R vs. NR	1.30	4.77 × 10^−2^	5.49 × 10^−1^	C vs. P	1.23	2.76 × 10^−2^	3.65 × 10^−1^
*TSPAN33*	Tetraspanin 33	R vs. NR	0.71	2.07 × 10^−2^	5.22 × 10^−1^	C vs. P	0.75	9.26 × 10^−3^	2.48 × 10^−1^
*ZNF844*	Zinc finger protein 844	R vs. NR	1.28	4.24 × 10^−2^	5.41 × 10^−1^	C vs. P	1.21	3.51 × 10^−2^	3.96 × 10^−1^

*C vs. P* controls vs. patients, *R vs. NR* responders vs. nonresponders, *Pre vs. Post* at baseline vs. at 6 weeks after antidepressant treatment, *FC* fold change, *SSRIs* selective serotonin reuptake inhibitors

^*a^Fold changes in patients compared to controls (P/C), at baseline in nonresponders compared to responders (NR/R), and at 6 weeks of antidepressant treatment compared to baseline (Post/Pre)

Comparing the results from GO enrichment and pathway analyses, one GO biological process term and one pathway were commonly obtained in comparisons between MDD patients and controls and between responders and nonresponders to SSRIs: immune response (GO:0006955) and signaling in the immune system (REACT_6900), respectively.

### Genes related to mirtazapine treatment response

We analyzed data from patients treated with mirtazapine in the same way that we did for patients treated with SSRIs. Two hundred eighty-one DEGs, 23 GO terms, and five pathways were identified in a comparison between mirtazapine responders and nonresponders (Supplementary Table 1). GO terms involved in coagulation and DNA damage response were the most enriched. One gene, *UBE2D3*, showed a change in expression according to mirtazapine treatment. The top 10 downregulated and upregulated genes were identified based on the FC values and are listed in Supplementary Table 4.

Among the identified DEGs, 21 genes were identified both in comparisons between MDD patients and controls and between responders and nonresponders to mirtazapine (Table 3). In GO and pathway enrichment analyses using the above 21 genes, three GO biological process terms were identified: response to DNA damage stimulus (GO:0006974), DNA damage checkpoint (GO:0000077), and DNA integrity checkpoint (GO:0031570).

## Discussion

In this study, we performed an RNA microarray analysis using peripheral blood specimens taken at baseline and at 6 weeks after antidepressant treatment in MDD patients. We identified genes and biological processes associated with MDD and with treatment response to antidepressants. We also obtained comparable results from public microarray data and from multiple comparisons performed in our study: between MDD patients and controls, between responders and nonresponders, and between baseline and 6 weeks after antidepressant treatment.

Genes involved in immune and inflammatory responses and apoptosis were among the most highly upregulated and downregulated in MDD: *BTNL8*, *CXCL8*, *LRIF1*, *NFKBIA*, *NLRC4*, *RGS1*, *RNASEL*, *TNFAIP3*, and *UBAP2L*. Similarly, among the seven DEGs that were commonly identified in both our study and the public microarray data, the genes *CD58*, *CXCL8*, *TARP*, and *TNFSF4* are also involved in immune and inflammatory responses. The *CXCL8* gene, which encodes interleukin-8 (IL-8), was among the 10 most highly downregulated genes in both our study and the public microarray data. Our previous study also showed a decrease in IL-8 concentration in MDD patients compared to healthy individuals^55^. Although *CXCL8* has not been proposed as a biomarker of MDD in previous gene expression studies, abnormalities in protein levels of pro-inflammatory cytokines, including IL-8, have been reported in MDD^56–59^, and alterations in the expression of genes related to cytokines other than IL-8 have also been observed in previous studies, e.g., alteration of *CCL24* gene expression in peripheral blood from MDD patients was identified using targeted gene expression analysis^60^. CD58, lymphocyte function-associated antigen 3, functions in the adhesion and activation of T lymphocytes^61^. Polymorphisms in the *CD58* gene have recently been reported to be related to the risk of multiple sclerosis through alterations in the processing of microRNA^62^. The protein encoded by the *TNFSF4* gene is also involved in adhesion of activated T lymphocytes. Although neither the *CD58* nor the *TNFSF4* gene has ever been studied at the DNA, RNA, or protein level in MDD, various microarray and targeted gene expression analyses have suggested that immune and inflammatory responses play a role in MDD^10,29,32,63–66^. These genes, including *CXCL8*, would be candidates for further studies evaluating the pathologic changes in MDD.

Similarly, GO and pathway enrichment analyses and network analysis also showed that most DEGs in MDD in our study and the public microarray data are involved in immune and inflammatory responses and apoptosis. Recent gene expression studies in MDD have also shown that immune suppression and immune activation would be associated with the etiology of MDD, based on GO and pathway enrichment analyses^38,67^. The role of immune and inflammatory responses in MDD has garnered interest;^10,11,32,68^ increased concentrations of cytokines and acute phase proteins in MDD^69,70^, induction of depressive-like symptoms by administration of pro-inflammatory cytokines^70^, and antidepressive effects of anti-inflammatory drugs have all been reported^12,70–74^. In the pathophysiology of MDD, alterations in the immune and inflammatory systems overlap and are involved in oxidative stress; mitochondrial dysfunction; neuroprogression, including neurodegeneration, neuronal apoptosis, and reduced neurogenesis; and serotonin metabolism disruption^10,11^. Altered gene expression related to these mechanisms in our study might be associated with immune and inflammatory responses in a broad sense. Epidermal growth factor, which is encoded by the *EGF* gene and was differentially expressed in both our study and in the public data, is a neurotrophic factor that plays a role in neurogenesis and neuronal plasticity^75–77^. Reduction in the levels of neurotrophic factors, including epidermal growth factor, in MDD has been reported in previous studies^78–80^. The *ERMN* and *LRRN3* genes, which have roles in neuroplasticity and neuronal development and maintenance^81^, were among the top 10 upregulated and downregulated genes in our study. Clinical research results on the relationships between these genes and MDD have not yet been reported, and the nature of alterations in immune and inflammatory responses has not been fully elucidated and might involve complex mechanisms^10,58^.

The genes involved in immune and inflammatory responses that were among the top 10 downregulated and upregulated genes related to responsiveness to SSRI treatment consisted of *BTNL8*, *CLC*, *CTSW*, *FCRL6*, *GNAQ*, *HLA-DPB1*, *IGKC*, *KIR2DS1*, *NOD2*, *USP41*, *VNN1*, and *XCL1*. The *BTNL8*, *HSPH1*, *IGKC*, *KATNBL1*, *LYVE1*, *MIR15A*, *PTCH2*, and *SCARNA17* genes were also found to be differently expressed between MDD patients and controls. The *BTNL8* gene, which plays an essential role in primary immune responses^82^, was among the top 10 downregulated and upregulated genes in both comparisons. In addition, the *FCRL6* gene was more highly expressed in responders than in nonresponders and was downregulated relative to the control group after SSRI treatment only in responders. The *FCRL6* gene encodes Fc receptor-like protein 6, which is involved in the interaction between cytotoxic lymphocytes and antigen-presenting cells (APCs) and is a member of the MHC class II receptor family^83^. Therefore, immune and inflammatory systems, including the *BTNL8* and *FCRL6* genes, might be involved in responsiveness to SSRIs as well as the underlying derangement in MDD. Previous transcriptomic studies have focused on the differences between MDD patients and healthy controls^32,63,65,66,84^, and there have been few clinical transcriptomic studies attempting to identify genetic biomarkers associated with antidepressant treatment response in MDD^27–31^. Our study findings on differences in gene expression according to treatment response and gene expression changes with antidepressant treatment have great implications for understanding and predicting treatment responses to antidepressants in MDD. In particular, our finding that the *BTNL8* and *FCRL6* genes have relatively large differences in expression in two comparisons (Table 3 and Supplementary Tables 2 and 3) has not been previously reported; accordingly, further validation studies will be required to confirm these findings.

Among the 281 genes that showed expression differences related to responsiveness to mirtazapine treatment, 21 were also differentially expressed between MDD patients and controls. In GO and pathway enrichment analyses of these 21 genes, GO terms involved in coagulation and DNA damage responses were identified. In the investigation of the 21 individual DEGs, the *SLA2* and *TOPORS* genes, which might be involved in immune responses and apoptosis, were identified, as well as the *CEP63* gene, which plays a role in the response to DNA damage. Disruption of DNA damage responses in MDD has previously been demonstrated in several studies^11–13^, including targeted gene expression analyses^16,17^. However, individual genes involved in DNA damage responses have not been previously identified in the context of MDD and antidepressant treatment. Our findings support a role for DNA damage as a result of immune activation in MDD, and the finding that the expression of these genes in responders to mirtazapine was closer to the expression levels of controls than to those of nonresponders implies that these genes and their mechanisms might be relevant to treatment resistance to mirtazapine.

In this study, we performed gene expression profiling and various comparison analyses. However, we should acknowledge the limitations of this study. We did not perform quantitative PCR, which has usually been employed in previous microarray studies, and we did not perform replicate analysis of each specimen. However, we validated our study results in other ways, including analysis of public microarray data, comparison with our cytokine study results^55^, and through replicates of comparisons that were performed in our study: between MDD patients and controls, between responders and nonresponders, and between baseline and 6 weeks after antidepressant treatment. Another limitation of our study involves the issues of study population and sample size. The lack of GO terms and pathways with statistically significant FDR-corrected p-values could be due to the limited sample size. Although our study population had a high proportion of older patients and females, MDD patients and healthy individuals did not show any clustering of baseline expression according to age and sex. The replication of our findings by future studies is needed to clarify and prove that the genes and mechanisms we identified are associated with MDD and responsiveness to antidepressant treatment. Our study has certain advantages in the discovery of genes related to antidepressant treatment response, which has rarely been a subject of transcriptomic studies, and in the classification of analyses according to type of antidepressant used. Most previous studies performed gene expression in MDD patients compared with healthy controls^85^. There have been few previous transcriptomic studies in MDD patients aiming to identify biomarkers related to antidepressant responsiveness^27–31^. Our study is the first to examine the treatment response to mirtazapine and to analyze changes in gene expression using genome-wide microarray techniques in responders compared to non-responders, and it is unique in that it was conducted in a non-Caucasian ethnic group^39^.

Various genes and mechanisms involved in immune and inflammatory responses, including the *CXCL8*, *BTNL8*, and *FCRL6* genes, which are commonly related to T lymphocytes, were identified in our study. However, as in most previous studies, we did not confirm whether these differences and changes are the causes or the results of a depressive episode. The mechanism underlying these differences and their relation to disease and treatment response remains unknown because our results are preliminary and have not been verified by functional analysis. MDD is a multifactorial disorder, and a previous study proposed that complex and multiple mechanisms, including the disruption of oxidative stress response, damage to DNA and mitochondria, and neuroprogression including neuronal apoptosis and lowered neuroplasticity, as concomitants and sequelae of the activation of immune and inflammatory systems play roles in the development of MDD^11^. Various genes involved in mechanisms other than those mentioned above also showed alterations in expression in our study. Our study results support the complexity of the development and treatment response of MDD. The complete interrelationships among these multiple mechanisms cannot be detected with the current statistical analyses; thus, the application and development of further bioinformatic analyses would be required to dissect these complex associations in MDD. In addition, it will be necessary to correlate these findings with other studies, including proteomic and metabolic results, to thoroughly investigate the roles of the identified genes and mechanisms in the development of MDD and antidepressant responses.

In summary, our study identified several interesting genes and mechanisms that might be associated with MDD and treatment responsiveness to antidepressants using RNA microarray analyses of peripheral blood specimens from MDD patients. In this respect, our study provides clinical evidence relevant to previous theories on the development of MDD and can serve as a foundation for future studies on antidepressant treatment response. Our study results support the proposition that the development of MDD and antidepressant responses are associated with a series of events that includes DNA damage and apoptosis stemming from immune and inflammatory activation in MDD. Our study is the first to analyze changes in gene expression using genome-wide microarray techniques in treatment responders compared to non-responders. Our findings contribute to the elucidation of the biological disturbance of MDD and might lead to early therapeutic intervention and personalized medicine for the treatment of MDD with antidepressants. Specifically, genes that are involved in immune and inflammatory responses and their sequelae, including *BTNL8*, *CXCL8*, and *FCRL6*, are candidates for prediction of antidepressant treatment response as well as for diagnosis of MDD.

## Electronic supplementary material

Supplementary table 1

Supplementary table 2

Supplementary table 3

Supplementary table 4
