# Supplementary material for: Differentially expressed genes related to major depressive disorder and antidepressant response: genome-wide gene expression analysis
Source: Exp Mol Med. 2018 Aug 3;50(8):92. doi: 10.1038/s12276-018-0123-0 (PMC6076250; doi:10.1038/s12276-018-0123-0)
Supplement: Supplementary file 1 — Supplementary table 1 [file 12276_2018_123_MOESM1_ESM.pdf]

Supplementary Table 1 Gene Ontology (GO) and pathway enrichment analyses

Category	Accession No.	GO term	Count No.[a]	Count %[b]	P [c]
MDD patients vs. controls					
GO-biological process	GO:0006350	Transcription	73	15.3%	0.000
Reactome pathway	REACT_6900	Signaling in Immune system	17	3.6%	0.000
GO-biological process	GO:0045449	Regulation of transcription	78	16.4%	0.002
GO-biological process	GO:0045321	Leukocyte activation	14	2.9%	0.003
GO-biological process	GO:0006955	Immune response	26	5.5%	0.009
GO-biological process	GO:0001775	Cell activation	14	2.9%	0.011
GO-biological process	GO:0046649	Lymphocyte activation	11	2.3%	0.012
GO-biological process	GO:0010717	Regulation of epithelial to mesenchymal transition	3	0.6%	0.016
GO-biological process	GO:0007243	Protein kinase cascade	16	3.4%	0.016
GO-biological process	GO:0016310	Phosphorylation	28	5.9%	0.017
GO-biological process	GO:0001816	Cytokine production	5	1.1%	0.018
GO-biological process	GO:0045637	Regulation of myeloid cell differentiation	6	1.3%	0.018
GO-biological process	GO:0034613	Cellular protein localization	17	3.6%	0.019
GO-biological process	GO:0070727	Cellular macromolecule localization	17	3.6%	0.020
GO-biological process	GO:0032886	Regulation of microtubule-based process	5	1.1%	0.022
GO-biological process	GO:0050821	Protein stabilization	4	0.8%	0.023
GO-biological process	GO:0045742	Positive regulation of epidermal growth factor receptor signaling pathway	3	0.6%	0.023
KEGG pathway	hsa04612	Antigen processing and presentation	6	1.3%	0.028
GO-biological process	GO:0043281	Regulation of caspase activity	6	1.3%	0.029
GO-biological process	GO:0008104	Protein localization	29	6.1%	0.030
KEGG pathway	hsa04210	Apoptosis	6	1.3%	0.033
GO-biological process	GO:0007049	Cell cycle	26	5.5%	0.034
GO-biological process	GO:0052548	Regulation of endopeptidase activity	6	1.3%	0.034
GO-biological process	GO:0051252	Regulation of RNA metabolic process	52	10.9%	0.034
GO-biological process	GO:0006355	Regulation of transcription, DNA-dependent	51	10.7%	0.034
GO-biological process	GO:0006886	Intracellular protein transport	15	3.2%	0.036
KEGG pathway	hsa05215	Prostate cancer	6	1.3%	0.036
GO-biological process	GO:0007167	Enzyme linked receptor protein signaling pathway	14	2.9%	0.038
GO-biological process	GO:0051100	Negative regulation of binding	5	1.1%	0.040
GO-biological process	GO:0052547	Regulation of peptidase activity	6	1.3%	0.040
GO-biological process	GO:0006915	Apoptosis	21	4.4%	0.042
GO-biological process	GO:0045785	Positive regulation of cell adhesion	5	1.1%	0.042
GO-biological process	GO:0010719	Negative regulation of epithelial to mesenchymal transition	2	0.4%	0.043
GO-biological process	GO:0015031	Protein transport	25	5.3%	0.046
GO-biological process	GO:0012501	Programmed cell death	21	4.4%	0.048
GO-biological process	GO:0007249	I-kappaB kinase/NF-kappaB cascade	5	1.1%	0.049
Responders vs. Nonresponders to SSRIs					
GO-biological process	GO:0051092	Positive regulation of NF-kappaB transcription factor activity	5	2.3%	0.001
GO-biological process	GO:0044275	Cellular carbohydrate catabolic process	6	2.7%	0.003
GO-biological process	GO:0006098	Pentose-phosphate shunt	3	1.4%	0.005
KEGG pathway	hsa00030	Pentose phosphate pathway	4	1.8%	0.005
GO-biological process	GO:0030036	Actin cytoskeleton organization	9	4.1%	0.005
GO-biological process	GO:0051091	Positive regulation of transcription factor activity	5	2.3%	0.005
GO-biological process	GO:0006955	Immune response	17	7.7%	0.007
KEGG pathway	hsa05216	Thyroid cancer	4	1.8%	0.008
GO-biological process	GO:0030029	Actin filament-based process	9	4.1%	0.008
GO-biological process	GO:0055114	Oxidation reduction	16	7.2%	0.008
Reactome pathway	REACT_6900	Signaling in Immune system	11	5.0%	0.009
GO-biological process	GO:0043388	Positive regulation of DNA binding	5	2.3%	0.009
GO-biological process	GO:0016052	Carbohydrate catabolic process	6	2.7%	0.009
GO-biological process	GO:0051099	Positive regulation of binding	5	2.3%	0.013
GO-biological process	GO:0007010	Cytoskeleton organization	12	5.4%	0.014
GO-biological process	GO:0032088	Negative regulation of NF-kappaB transcription factor activity	3	1.4%	0.015
GO-biological process	GO:0006739	NADP metabolic process	3	1.4%	0.015
GO-biological process	GO:0043067	Regulation of programmed cell death	18	8.1%	0.015
GO-biological process	GO:0010941	Regulation of cell death	18	8.1%	0.015
GO-biological process	GO:0007242	Intracellular signaling cascade	24	10.8%	0.021
GO-biological process	GO:0009051	Pentose-phosphate shunt, oxidative branch	2	0.9%	0.023
GO-biological process	GO:0045893	Positive regulation of transcription, DNA-dependent	12	5.4%	0.025
GO-biological process	GO:0051254	Positive regulation of RNA metabolic process	12	5.4%	0.027
GO-biological process	GO:0042981	Regulation of apoptosis	17	7.7%	0.027
GO-biological process	GO:0031328	Positive regulation of cellular biosynthetic process	15	6.8%	0.031
GO-biological process	GO:0045944	Positive regulation of transcription from RNA polymerase II promoter	10	4.5%	0.031
GO-biological process	GO:0051090	Regulation of transcription factor activity	5	2.3%	0.033
GO-biological process	GO:0045941	Positive regulation of transcription	13	5.9%	0.034
GO-biological process	GO:0009891	Positive regulation of biosynthetic process	15	6.8%	0.034
GO-biological process	GO:0006006	Glucose metabolic process	6	2.7%	0.034
GO-biological process	GO:0019322	Pentose biosynthetic process	2	0.9%	0.035
GO-biological process	GO:0051173	Positive regulation of nitrogen compound metabolic process	14	6.3%	0.040
GO-biological process	GO:0010628	Positive regulation of gene expression	13	5.9%	0.041

Supplementary Table 1 Gene Ontology (GO) and pathway enrichment analyses

Category	Accession No.	GO term	Count No.[a]	Count %[b]	*P* [c]
GO-biological process	GO:0007507	Heart development	7	3.2%	0.042
GO-biological process	GO:0044093	Positive regulation of molecular function	13	5.9%	0.044
GO-biological process	GO:0010604	Positive regulation of macromolecule metabolic process	17	7.7%	0.045
Responders vs. Nonresponders to mirtazapine					
GO-biological process	GO:0007242	Intracellular signaling cascade	31	11.0%	0.001
GO-biological process	GO:0007596	Blood coagulation	7	2.5%	0.002
GO-biological process	GO:0050817	Coagulation	7	2.5%	0.002
GO-biological process	GO:0007599	Hemostasis	7	2.5%	0.003
Panther pathway	P00011	Blood coagulation	5	1.8%	0.008
GO-biological process	GO:0030168	Platelet activation	4	1.4%	0.008
GO-biological process	GO:0050878	Regulation of body fluid levels	7	2.5%	0.011
GO-biological process	GO:0030193	Regulation of blood coagulation	4	1.4%	0.011
GO-biological process	GO:0042060	Wound healing	8	2.8%	0.013
GO-biological process	GO:0006937	Regulation of muscle contraction	5	1.8%	0.015
GO-biological process	GO:0050818	Regulation of coagulation	4	1.4%	0.016
KEGG pathway	hsa04020	Calcium signaling pathway	7	2.5%	0.017
GO-biological process	GO:0042770	DNA damage response, signal transduction	5	1.8%	0.021
GO-biological process	GO:0007155	Cell adhesion	17	6.0%	0.021
GO-biological process	GO:0022610	Biological adhesion	17	6.0%	0.022
GO-biological process	GO:0045987	Positive regulation of smooth muscle contraction	3	1.1%	0.023
GO-biological process	GO:0000077	DNA damage checkpoint	4	1.4%	0.025
KEGG pathway	hsa04666	Fc gamma R-mediated phagocytosis	5	1.8%	0.025
GO-biological process	GO:0045933	Positive regulation of muscle contraction	3	1.1%	0.030
GO-biological process	GO:0031570	DNA integrity checkpoint	4	1.4%	0.030
GO-biological process	GO:0000075	Cell cycle checkpoint	5	1.8%	0.031
GO-biological process	GO:0030195	Negative regulation of blood coagulation	3	1.1%	0.036
GO-biological process	GO:0048771	Tissue remodeling	4	1.4%	0.037
Reactome pathway	REACT_604	Hemostasis	8	2.8%	0.040
KEGG pathway	hsa04270	Vascular smooth muscle contraction	5	1.8%	0.042
GO-biological process	GO:0042325	Regulation of phosphorylation	12	4.3%	0.043
GO-biological process	GO:0050819	Negative regulation of coagulation	3	1.1%	0.045
GO-biological process	GO:0048146	Positive regulation of fibroblast proliferation	3	1.1%	0.045

[a]The number of genes that have a corresponding GO term or pathway

[b]The proportion of genes that have a corresponding GO term or pathway among the total DEGs in each comparison (p-values < 0.05 and absolute fold change > 1.2)

[c]Uncorrected p-values
