# Supplementary material for: Differentially expressed genes related to major depressive disorder and antidepressant response: genome-wide gene expression analysis
Source: Exp Mol Med. 2018 Aug 3;50(8):92. doi: 10.1038/s12276-018-0123-0 (PMC6076250; doi:10.1038/s12276-018-0123-0)
Supplement: Supplementary file 2 — Supplementary table 2 [file 12276_2018_123_MOESM2_ESM.pdf]

Supplementary Table 2 Top 10 downregulated and upregulated genes in major depressive disorder patients compared to healthy controls

Gene symbol	Full gene name	Associated GO term[a]	FC[b]	*P*	Corrected *P*
SNORD41	Small nucleolar RNA, C/D box 41	NA	0.54	3.54×10^{-5}	3.33×10^{-2}
ERMN	Ermin, ERM-like protein	Actin filament organization; morphogenesis of a branching structure; regulation of cell projection organization; regulation of cell shape	0.60	7.94×10^{-5}	5.20×10^{-2}
PTCH2	Patched 2	Cell fate determination; epidermal cell fate specification; hair cycle; negative regulation of smoothened signaling pathway; positive regulation of epidermal cell differentiation; signal transduction; skin development	0.60	7.97×10^{-4}	1.04×10^{-1}
RGS1	Regulator of G-protein signaling 1	Adenylate cyclase-inhibiting G-protein coupled receptor signaling pathway; immune response; negative regulation of signal transduction; positive regulation of GTPase activity; regulation of G-protein coupled receptor protein signaling pathway; signal transduction	0.65	1.39×10^{-2}	2.92×10^{-1}
TNFAIP3	Tumor necrosis factor, alpha-induced protein 3	Apoptotic process; B-1 B cell homeostasis; cell surface receptor signaling pathway; cellular response to hydrogen peroxide; cellular response to lipopolysaccharide; establishment of protein localization to vacuole; immune response; inflammatory response; innate immune response; negative regulation of B cell activation; negative regulation of bone resorption; negative regulation of CD40 signaling pathway; negative regulation of chronic inflammatory response; negative regulation of cyclin-dependent protein serine/threonine kinase activity; negative regulation of endothelial cell apoptotic process; negative regulation of extrinsic apoptotic signaling pathway via death domain receptors; negative regulation of I-kappaB kinase/NF-kappaB signaling; negative regulation of inflammatory response; negative regulation of innate immune response; negative regulation of interleukin-1 beta production; negative regulation of interleukin-2 production; negative regulation of interleukin-6 production; negative regulation of NF-kappaB transcription factor activity; negative regulation of nucleotide-binding oligomerization domain containing 1/2 signaling pathway; regulation of tumor necrosis factor-mediated signaling pathway; regulation of vascular wound healing; response to molecule of bacterial origin; response to muramyl dipeptide; tolerance induction to lipopolysaccharide; tumor necrosis factor-mediated signaling pathway	0.67	7.15×10^{-3}	2.25×10^{-1}
FAM111B	Family with sequence similarity 111, member B	NA	0.68	3.24×10^{-3}	1.70×10^{-1}
NFKBIA	Nuclear factor of kappa light polypeptide gene enhancer in B-cells inhibitor, alpha	Apoptotic process; cellular response to cold; cytoplasmic sequestering of NF-kappaB; cytoplasmic sequestering of transcription factor; Fc-epsilon receptor signaling pathway; innate immune response; lipopolysaccharide-mediated signaling pathway; MyD88-dependent/independent toll-like receptor signaling pathway; negative regulation of apoptotic process; negative regulation of DNA binding; negative regulation of lipid storage; negative regulation of macrophage derived foam cell differentiation; negative regulation of myeloid cell differentiation; negative regulation of NF-kappaB transcription factor activity; negative regulation of Notch signaling pathway; neurotrophin TRK receptor signaling pathway; nucleotide-binding oligomerization domain containing 1/2 signaling pathway; positive regulation of cellular protein metabolic process; positive regulation of cholesterol efflux; positive regulation of NF-kappaB transcription factor activity; positive regulation of transcription from RNA polymerase II promoter; positive regulation of type I interferon production; protein import into nucleus, translocation; regulation of cell proliferation; regulation of NF-kappaB import into nucleus; response to exogenous dsRNA; response to muramyl dipeptide; response to muscle stretch; stimulatory C-type lectin receptor signaling pathway; T cell receptor signaling pathway; toll-like receptor 2/3/4/5/9/10 signaling pathway; toll-like receptor signaling pathway; toll-like receptor TLR1:TLR2 signaling pathway; toll-like receptor TLR6:TLR2 signaling pathway; TRIF-dependent toll-like receptor signaling pathway; viral process	0.68	4.50×10^{-3}	1.92×10^{-1}
UBAP2L	Ubiquitin associated protein 2-like	Binding of sperm to zona pellucida; hematopoietic stem cell homeostasis	0.69	5.22×10^{-5}	4.44×10^{-2}
CXCL8	Chemokine (C-X-C motif) ligand 8	Angiogenesis; calcium-mediated signaling; cell cycle arrest; cellular protein metabolic process; cellular response to fibroblast growth factor stimulus; cellular response to interleukin-1; cellular response to lipopolysaccharide; cellular response to tumor necrosis factor; chemokine-mediated signaling pathway; chemotaxis; embryonic digestive tract development; endoplasmic reticulum unfolded protein response; G-protein coupled receptor signaling pathway; immune response; induction of positive chemotaxis; inflammatory response; intracellular signal transduction; movement of cell or subcellular component; negative regulation of cell proliferation; negative regulation of G-protein coupled receptor protein signaling pathway; neutrophil activation; neutrophil chemotaxis; PERK-mediated unfolded protein response; positive regulation of angiogenesis; positive regulation of neutrophil chemotaxis; receptor internalization; regulation of cell adhesion; regulation of single stranded viral RNA replication via double stranded DNA intermediate; response to endoplasmic reticulum stress; response to molecule of bacterial origin; signal transduction	0.71	3.45×10^{-2}	3.93×10^{-1}
EIF4H	Eukaryotic translation initiation factor 4H	Cellular protein metabolic process; gene expression; regulation of translational initiation; translation; translational initiation; viral process	0.71	3.08×10^{-4}	7.60×10^{-2}
NLRC4	NLR family, CARD domain containing 4	Activation of cysteine-type endopeptidase activity involved in apoptotic process; activation of innate immune response; defense response to bacterium; detection of bacterium; inflammatory response; inhibition of cysteine-type endopeptidase activity involved in apoptotic process; innate immune response; interleukin-1 beta secretion; mitotic spindle assembly; nucleotide-binding domain, leucine rich repeat containing receptor signaling pathway; positive regulation of apoptotic process; positive regulation of NF-kappaB transcription factor activity; protein homooligomerization; protein ubiquitination; pyroptosis; regulation of signal transduction	1.55	6.41×10^{-3}	2.17×10^{-1}
GOLPH3L	Golgi phosphoprotein 3-like	Golgi organization; Golgi vesicle transport; positive regulation of protein secretion	1.56	2.54×10^{-6}	1.37×10^{-2}
RNASEL	Ribonuclease L (2',5'-oligoisoadenylate synthetase-dependent)	Cytokine-mediated signaling pathway; defense response to virus; fat cell differentiation; mRNA processing; negative regulation of viral genome replication; positive regulation of glucose import in response to insulin stimulus; positive regulation of transcription from RNA polymerase II promoter; protein phosphorylation; regulation of mRNA stability; RNA phosphodiester bond hydrolysis, endonucleolytic; rRNA processing; transmembrane transport; type I interferon signaling pathway	1.56	1.02×10^{-4}	5.21×10^{-2}
BTNL8	Butyrophilin-like 8	Adaptive immune response	1.59	1.63×10^{-3}	1.33×10^{-1}
LINC00189	Long intergenic non-protein coding RNA 189	NA	1.62	4.98×10^{-2}	4.50×10^{-1}
LINC00266-1	Long intergenic non-protein coding RNA 266-1	NA	1.64	3.86×10^{-4}	8.15×10^{-2}
LRIF1	Ligand dependent nuclear receptor interacting factor 1	Regulation of transcription, DNA-templated; transcription, DNA-templated	1.65	4.72×10^{-6}	1.60×10^{-2}
CEP19	Centrosomal protein 19kDa	NA	1.71	1.80×10^{-3}	1.39×10^{-1}
LRRN3	Leucine rich repeat neuronal 3	Positive regulation of synapse assembly	1.72	6.78×10^{-3}	2.22×10^{-1}
KBTBD7	Kelch repeat and BTB (POZ) domain containing 7	Activation of MAPKK activity; axon guidance; epidermal growth factor receptor signaling pathway; Fc-epsilon receptor signaling pathway; fibroblast growth factor receptor signaling pathway; innate immune response; insulin receptor signaling pathway; MAPK cascade; neurotrophin TRK receptor signaling pathway; protein ubiquitination; Ras protein signal transduction; small GTPase mediated signal transduction; vascular endothelial growth factor receptor signaling pathway	1.77	2.20×10^{-4}	6.58×10^{-2}

FC: fold change; GO: Gene Ontology; NA: not available.

[a]Gene Ontology biologic process terms

[b]Fold changes in patients compared to controls.
