# Supplementary material for: Differentially expressed genes related to major depressive disorder and antidepressant response: genome-wide gene expression analysis
Source: Exp Mol Med. 2018 Aug 3;50(8):92. doi: 10.1038/s12276-018-0123-0 (PMC6076250; doi:10.1038/s12276-018-0123-0)
Supplement: Supplementary file 3 — Supplementary table 3 [file 12276_2018_123_MOESM3_ESM.pdf]

Supplementary Table 3 Top 10 downregulated and upregulated genes related to selective serotonin reuptake inhibitor responsiveness

Gene symbol	Full gene name	Associated GO term[a]	FC	*P*	Corrected *P*
Responders vs. Nonresponders to SSRIs[b]					
KIAA1324	KIAA1324	Cellular response to starvation; macroautophagy; negative regulation of extrinsic apoptotic signaling pathway in absence of ligand; positive regulation of autophagosome assembly; positive regulation of vacuole organization	0.44	1.90×10^{-2}	4.27×10^{-1}
CLC	Charcot-Leyden crystal galectin	Multicellular organismal development; regulation of activated T cell proliferation; regulation of T cell anergy; regulation of T cell cytokine production; T cell apoptotic process	0.48	3.83×10^{-2}	4.84×10^{-1}
NOD2	Nucleotide-binding oligomerization domain containing 2	Activation of MAPK activity; activation of MAPK activity involved in innate immune response; cellular response to muramyl dipeptide; cellular response to peptidoglycan; cytokine production involved in immune response; cytokine secretion involved in immune response; defense response; defense response to bacterium; defense response to Gram-positive bacterium; detection of bacterium; detection of biotic stimulus; detection of muramyl dipeptide; immunoglobulin production involved in immunoglobulin mediated immune response; innate immune response; innate immune response in mucosa; intracellular signal transduction; JNK cascade; macrophage inflammatory protein-1 alpha production; maintenance of gastrointestinal epithelium; MyD88-dependent toll-like receptor signaling pathway; MyD88-independent toll-like receptor signaling pathway; negative regulation of growth of symbiont in host; negative regulation of inflammatory response to antigenic stimulus; negative regulation of interferon-gamma production; negative regulation of interleukin-2/12/18 production; negative regulation of macrophage apoptotic process; negative regulation of NF-kappaB transcription factor activity; negative regulation of T cell mediated immunity; negative regulation of toll-like receptor 2 signaling pathway; negative regulation of tumor necrosis factor production; nucleotide-binding domain, leucine rich repeat containing receptor signaling pathway; nucleotide-binding oligomerization domain containing 2 signaling pathway; nucleotide-binding oligomerization domain containing signaling pathway; positive regulation of B cell activation; positive regulation of biosynthetic process of antibacterial peptides active against Gram-positive bacteria; positive regulation of cytokine production involved in inflammatory response; positive regulation of dendritic cell antigen processing and presentation; positive regulation of epithelial cell proliferation; positive regulation of ERK1 and ERK2 cascade; positive regulation of gamma-delta T cell activation; positive regulation of humoral immune response mediated by circulating immunoglobulin; positive regulation of I-kappaB kinase/NF-kappaB signaling; positive regulation of interleukin-1 beta/6/8/10/12/17 production; positive regulation of interleukin-1 beta secretion; positive regulation of JNK cascade; positive regulation of NF-kappaB transcription factor activity; positive regulation of NIK/NF-kappaB signaling; positive regulation of nitric-oxide synthase biosynthetic process; positive regulation of Notch signaling pathway; positive regulation of oxidoreductase activity; positive regulation of peptidyl-tyrosine phosphorylation; positive regulation of phagocytosis; positive regulation of phosphatidylinositol 3-kinase activity; positive regulation of prostaglandin-E synthase activity; positive regulation of prostaglandin-endoperoxide synthase activity; positive regulation of stress-activated MAPK cascade; positive regulation of transcription from RNA polymerase II promoter; positive regulation of tumor necrosis factor production; positive regulation of type 2 immune response; protein oligomerization; regulation of inflammatory response; regulation of neutrophil chemotaxis; response to exogenous dsRNA; response to lipopolysaccharide; response to muramyl dipeptide; stress-activated MAPK cascade; toll-like receptor 2/3/4/5/9/10 signaling pathway; toll-like receptor signaling pathway; toll-like receptor TLR1:TLR2 signaling pathway; toll-like receptor TLR6:TLR2 signaling pathway; TRIF-dependent toll-like receptor signaling pathway	0.56	4.38×10^{-4}	3.80×10^{-1}
MIRLET7G	MicroRNA let-7g	NA	0.60	7.96×10^{-3}	4.01×10^{-1}
DSC2	Desmocollin 2	Bundle of His cell-Purkinje myocyte adhesion involved in cell communication; cardiac muscle cell-cardiac muscle cell adhesion; cell adhesion; homophilic cell adhesion via plasma membrane adhesion molecules; regulation of heart rate by cardiac conduction; regulation of ventricular cardiac muscle cell action potential	0.6	4.78×10^{-2}	5.05×10^{-1}
SEC14L1	SEC14-like 1 (S. cerevisiae)	Transport	0.61	1.45×10^{-2}	4.19×10^{-1}
PHOSPHO1	Phosphatase, orphan 1	Bone mineralization involved in bone maturation; dephosphorylation; endochondral ossification; glycerophospholipid biosynthetic process; phosphatidylcholine biosynthetic process; phosphatidylethanolamine biosynthetic process; phospholipid metabolic process; regulation of bone mineralization; small molecule metabolic process	0.62	3.65×10^{-2}	4.78×10^{-1}
NUAK2	NUAK family, SNF1-like kinase, 2	Actin cytoskeleton organization; apoptotic process; cellular response to glucose starvation; intracellular signal transduction; negative regulation of apoptotic process; protein phosphorylation	0.62	1.57×10^{-3}	3.92×10^{-1}
GNAQ	Guanine nucleotide binding protein (G protein), q polypeptide	Action potential; activation of phospholipase C activity; adenylate cyclase-activating G-protein coupled receptor signaling pathway; blood coagulation; developmental pigmentation; embryonic digit morphogenesis; entrainment of circadian clock; forebrain neuron development; glutamate receptor signaling pathway; G-protein coupled acetylcholine receptor signaling pathway; heart development; maternal behavior; metabolic process; negative regulation of protein kinase activity; neuron remodeling; phospholipase C-activating dopamine receptor signaling pathway; phototransduction, visible light; platelet activation; positive regulation of GTPase activity; post-embryonic development; protein stabilization; regulation of catenin import into nucleus; regulation of melanocyte differentiation; skeletal system development	0.63	7.61×10^{-4}	3.80×10^{-1}
ZNF586	Zinc finger protein 586	Regulation of transcription, DNA-templated; transcription, DNA-templated	0.64	5.50×10^{-4}	3.80×10^{-1}
LINC00965	Long intergenic non-protein coding RNA 965	NA	1.42	2.65×10^{-3}	3.92×10^{-1}
KIAA1324L	KIAA1324-like	NA	1.42	4.60×10^{-2}	5.03×10^{-1}
FCRL6	Fc receptor-like 6	NA	1.48	3.54×10^{-2}	4.78×10^{-1}
CTSW	Cathepsin W	Immune response; proteolysis involved in cellular protein catabolic process	1.50	2.40×10^{-2}	4.45×10^{-1}
KIR2DS1	Killer cell immunoglobulin-like receptor, two domains, short cytoplasmic tail, 1	Immune response; innate immune response; regulation of immune response; signal transduction	1.54	3.27×10^{-2}	4.71×10^{-1}
GOLGA8A	Golgin A8 family, member A	NA	1.58	1.21×10^{-2}	4.16×10^{-1}

Supplementary Table 3 Top 10 downregulated and upregulated genes related to selective serotonin reuptake inhibitor responsiveness

Gene symbol	Full gene name	Associated GO term[a]	FC	*P*	Corrected *P*
XCL1	Chemokine (C motif) ligand 1	Cell-cell signaling; cellular response to interferon-gamma; cellular response to interleukin-1/4; cellular response to transforming growth factor beta stimulus; cellular response to tumor necrosis factor; chemokine-mediated signaling pathway; G-protein coupled receptor signaling pathway; inflammatory response; mature natural killer cell chemotaxis; monocyte chemotaxis; negative regulation of CD4-positive, alpha-beta T cell proliferation; negative regulation of interferon-gamma production; negative regulation of interleukin-2 production; negative regulation of sequence-specific DNA binding transcription factor activity; negative regulation of T cell cytokine production; negative regulation of T-helper 1 cell activation; negative regulation of T-helper 1 type immune response; negative regulation of transcription, DNA-templated; neutrophil chemotaxis; positive regulation of B cell chemotaxis; positive regulation of CD4-positive, alpha-beta T cell proliferation; positive regulation of CD8-positive, alpha-beta T cell proliferation; positive regulation of ERK1 and ERK2 cascade; positive regulation of granzyme A/B production; positive regulation of GTPase activity; positive regulation of immunoglobulin production in mucosal tissue; positive regulation of inflammatory response; positive regulation of interleukin-10 production; positive regulation of leukocyte chemotaxis; positive regulation of natural killer cell chemotaxis; positive regulation of neutrophil chemotaxis; positive regulation of release of sequestered calcium ion into cytosol; positive regulation of T cell chemotaxis; positive regulation of T cell cytokine production; positive regulation of T cell mediated cytotoxicity; positive regulation of T-helper 1/2 cell cytokine production; positive regulation of thymocyte migration; positive regulation of transforming growth factor beta production; regulation of inflammatory response; release of sequestered calcium ion into cytosol; response to virus; signal transduction	1.59	5.11×10^{-3}	3.92×10^{-1}
IGKC	Immunoglobulin kappa constant	B cell receptor signaling pathway; complement activation; complement activation, classical pathway; defense response to bacterium; Fc-epsilon receptor signaling pathway; Fc-gamma receptor signaling pathway involved in phagocytosis; immune response; innate immune response; phagocytosis, engulfment; phagocytosis, recognition; positive regulation of B cell activation; receptor-mediated endocytosis; regulation of immune response; retina homeostasis	1.64	6.30×10^{-3}	3.95×10^{-1}
GPRC5D	G protein-coupled receptor, class C, group 5, member D	G-protein coupled receptor signaling pathway	1.69	1.77×10^{-3}	3.92×10^{-1}
FAM118A	Family with sequence similarity 118, member A	NA	1.99	1.47×10^{-4}	3.80×10^{-1}
At baseline vs. after 6 weeks after SSRIs treatment only in responders[c]					
ARG1	Arginase 1	Aging; arginine catabolic process; arginine catabolic process to ornithine; cellular nitrogen compound metabolic process; cellular response to dexamethasone stimulus; cellular response to glucagon stimulus; cellular response to hydrogen peroxide; cellular response to interleukin-4; cellular response to lipopolysaccharide; cellular response to transforming growth factor beta stimulus; collagen biosynthetic process; liver development; lung development; mammary gland involution; maternal process involved in female pregnancy; polyamine metabolic process; positive regulation of endothelial cell proliferation; protein homotrimerization; regulation of L-arginine import; response to amine; response to amino acid; response to axon injury; response to cadmium ion; response to drug; response to herbicide; response to manganese ion; response to methylmercury; response to selenium ion; response to vitamin A/E; response to zinc ion; small molecule metabolic process; urea cycle	0.81	8.74×10^{-4}	5.94×10^{-2}
SULT1B1	Sulfotransferase family, cytosolic, 1B, member 1	3'-phosphoadenosine 5'-phosphosulfate metabolic process; cellular biogenic amine metabolic process; epithelial cell differentiation; flavonoid metabolic process; phenol-containing compound metabolic process; small molecule metabolic process; steroid metabolic process; sulfation; thyroid hormone metabolic process; xenobiotic metabolic process	0.81	3.71×10^{-3}	9.23×10^{-2}
BTNL8	Butyrophilin-like 8	Adaptive immune response	0.81	5.54×10^{-3}	1.06×10^{-1}
MIR15A	MicroRNA 15a	NA	0.81	2.82×10^{-2}	2.05×10^{-1}
TPST1	Tyrosylprotein sulfotransferase 1	Inflammatory response; peptidyl-tyrosine sulfation	0.81	1.03×10^{-2}	1.36×10^{-1}
LOC100131131	AHPA9419	NA	0.81	1.91×10^{-3}	7.14×10^{-2}
LIPN	Lipase, family member N	Lipid catabolic process	0.82	2.34×10^{-2}	1.89×10^{-1}
VNN1	Vanin 1	Acute inflammatory response; biotin metabolic process; central nervous system development; chronic inflammatory response; inflammatory response; innate immune response; movement of cell or subcellular component; negative regulation of oxidative stress-induced intrinsic apoptotic signaling pathway; pantothenate metabolic process; positive regulation of T cell differentiation in thymus; response to oxidative stress; single organismal cell-cell adhesion	0.82	1.60×10^{-3}	6.78×10^{-2}
MANSC1	MANSC domain containing 1	NA	0.82	4.63×10^{-2}	2.54×10^{-1}
FBXL13	F-box and leucine-rich repeat protein 13	NA	0.83	6.04×10^{-3}	1.09×10^{-1}
SPATS2L	Spermatogenesis associated, serine-rich 2-like	NA	1.21	4.91×10^{-3}	1.01×10^{-1}
FCRL6	Fc receptor-like 6	NA	1.21	1.72×10^{-3}	6.94×10^{-2}
SCARNA17	Small Cajal body-specific RNA 17	NA	1.21	1.08×10^{-3}	6.15×10^{-2}
SNORA80E	Small nucleolar RNA, H/ACA box 80E	NA	1.21	3.51×10^{-2}	2.26×10^{-1}
LOC642947	Uncharacterized LOC642947	NA	1.21	3.74×10^{-3}	9.24×10^{-2}
HLA-DPB1	Major histocompatibility complex, class II, DP beta 1	Antigen processing and presentation of exogenous peptide antigen via MHC class II; cytokine-mediated signaling pathway; interferon-gamma-mediated signaling pathway; positive regulation of interferon-gamma production; positive regulation of T cell activation; positive regulation of T cell proliferation; T cell costimulation; T cell receptor signaling pathway	1.22	1.04×10^{-3}	6.12×10^{-2}
MYOF	Myoferlin	Blood circulation; cellular response to heat; muscle contraction; plasma membrane repair; regulation of vascular endothelial growth factor receptor signaling pathway	1.23	9.93×10^{-4}	6.06×10^{-2}
USP41	Ubiquitin specific peptidase 41	Protein deubiquitination; ubiquitin-dependent protein catabolic process	1.23	1.63×10^{-3}	6.86×10^{-2}
MOP-1	MOP-1	NA	1.24	1.55×10^{-2}	1.60×10^{-1}
RNU5D-1	RNA, U5D small nuclear 1	NA	1.34	2.26×10^{-3}	7.61×10^{-2}

FC: fold change; GO: Gene Ontology; NA: not available; SSRIs: selective serotonin reuptake inhibitors.

[a]Gene Ontology biologic process terms

[b]Fold changes at baseline in nonresponders compared to responders.

[c]Fold changes in 6 weeks compared to baseline after SSRIs treatment.
