# Supplementary material for: Differentially expressed genes related to major depressive disorder and antidepressant response: genome-wide gene expression analysis
Source: Exp Mol Med. 2018 Aug 3;50(8):92. doi: 10.1038/s12276-018-0123-0 (PMC6076250; doi:10.1038/s12276-018-0123-0)
Supplement: Supplementary file 4 — Supplementary table 4 [file 12276_2018_123_MOESM4_ESM.pdf]

Supplementary Table 4 Top 10 downregulated and upregulated genes related to mirtazapine responsiveness

Gene symbol	Full gene name	Associated GO term[a]	FC[b]	*P*	Corrected *P*
MMRN1	Multimerin 1	Blood coagulation; cell adhesion; platelet activation; platelet degranulation	0.54	1.05×10^{-2}	5.07×10^{-1}
FAXDC2	Fatty acid hydroxylase domain containing 2	Fatty acid biosynthetic process; oxidation-reduction process	0.61	2.31×10^{-2}	5.26×10^{-1}
LGALS2	Lectin, galactoside-binding, soluble, 2	NA	0.61	1.43×10^{-2}	5.07×10^{-1}
CMTM5	CKLF-like MARVEL transmembrane domain containing 5	Chemotaxis; negative regulation of myoblast differentiation	0.62	1.31×10^{-2}	5.07×10^{-1}
RAB27B	RAB27B, member RAS oncogene family	Melanosome transport; metabolic process; multivesicular body sorting pathway; positive regulation of exocytosis; protein transport; small GTPase mediated signal transduction	0.62	3.32×10^{-2}	5.33×10^{-1}
GP1BA	Hlycoprotein Ib (platelet), alpha polypeptide	Blood coagulation; blood coagulation, intrinsic pathway; cell adhesion; cell morphogenesis; cell surface receptor signaling pathway; cytokine-mediated signaling pathway; fibrinolysis; negative regulation of cyclin-dependent protein kinase activity; negative regulation of JAK-STAT cascade; negative regulation of protein kinase activity; platelet activation; regulation of blood coagulation; thrombin receptor signaling pathway	0.62	3.32×10^{-2}	5.33×10^{-1}
TREML1	Triggering receptor expressed on myeloid cells-like 1	Calcium-mediated signaling; innate immune response; platelet activation; platelet activation; regulation of immune response	0.63	4.88×10^{-2}	5.49×10^{-1}
MYCT1	Myc target 1	NA	0.65	4.05×10^{-2}	5.37×10^{-1}
GUCY1B3	Guanylate cyclase 1, soluble, beta 3	Blood circulation; blood coagulation; cellular response to nitric oxide; cGMP biosynthetic process; nitric oxide mediated signal transduction; nitric oxide-cGMP-mediated signaling pathway	0.65	3.74×10^{-2}	5.37×10^{-1}
MYL9	Myosin, light chain 9, regulatory	Axon guidance; ephrin receptor signaling pathway; muscle contraction; platelet aggregation; regulation of muscle contraction; small GTPase mediated signal transduction	0.67	3.04×10^{-2}	5.26×10^{-1}
CCDC15	Coiled-coil domain containing 15	NA	1.52	4.01×10^{-4}	5.05×10^{-1}
TTTY10	Testis-specific transcript, Y-linked 10	NA	1.52	4.67×10^{-2}	5.48×10^{-1}
DAPK2	Death-associated protein kinase 2	Anoikis; apoptotic process; intracellular signal transduction; positive regulation of eosinophil chemotaxis; positive regulation of neutrophil chemotaxis; protein autophosphorylation; protein phosphorylation; regulation of apoptotic process; regulation of autophagy; regulation of intrinsic apoptotic signaling pathway	1.55	7.80×10^{-3}	5.05×10^{-1}
MIR646HG	MIR646 host gene	NA	1.58	2.77×10^{-3}	5.05×10^{-1}
NSUN7	NOP2/Sun domain family, member 7	Methylation	1.58	2.80×10^{-2}	5.26×10^{-1}
FMN1	Formin 1	Actin nucleation; dendrite morphogenesis; forelimb morphogenesis; gene expression; hindlimb morphogenesis; positive regulation of actin nucleation; positive regulation of focal adhesion assembly; skeletal system morphogenesis; ureteric bud invasion	1.62	1.76×10^{-2}	5.07×10^{-1}
MIR15B	MicroRNA 15b	NA	1.64	8.87×10^{-4}	5.05×10^{-1}
GRAMD1C	GRAM domain containing 1C	NA	1.65	2.47×10^{-2}	5.26×10^{-1}
FAM19A2	Family with sequence similarity 19 (chemokine (C-C motif)-like), member A2	NA	1.71	2.17×10^{-3}	5.05×10^{-1}
SNORD20	Small nucleolar RNA, C/D box 20	NA	1.88	2.53×10^{-3}	5.05×10^{-1}

FC: fold change; GO: Gene Ontology; NA: not available.

[a]Gene Ontology biologic process terms

[b]Fold changes in patients compared to controls.
